# The iodide transporter *Slc26a7* impacts thyroid function more strongly than *Slc26a4* in mice

**DOI:** 10.1038/s41598-022-15151-4

**Published:** 2022-07-04

**Authors:** Naoya Yamaguchi, Atsushi Suzuki, Aya Yoshida, Tatsushi Tanaka, Kohei Aoyama, Hisashi Oishi, Yuichiro Hara, Tomoo Ogi, Izuki Amano, Satomi Kameo, Noriyuki Koibuchi, Yasuhiro Shibata, Shinya Ugawa, Haruo Mizuno, Shinji Saitoh

**Affiliations:** 1 Department of Pediatrics and Neonatology, Nagoya City University Graduate School of Medical Sciences, 1-Kawasumi, Mizuho-cho, Mizuho-ku, Nagoya, 467-8601 Japan; 2 Department of Pediatrics, Toyohashi Municipal Hospital, Toyohashi, Japan; 3 Department of Comparative and Experimental Medicine, Nagoya City University Graduate School of Medical Sciences, Nagoya, Japan; 4 Department of Genetics, Research Institute of Environmental Medicine, Nagoya University, Nagoya, Japan; 5 Department of Human Genetics and Molecular Biology, Nagoya University Graduate School of Medicine, Nagoya, Japan; 6 Department of Integrative Physiology, Gunma University Graduate School of Medicine, Maebashi, Japan; 7 Department of Nutrition, Koshien University, Takarazuka, Japan; 8 Department of Anatomy and Neuroscience, Nagoya City University Graduate School of Medical Sciences, Nagoya, Japan; 9 Department of Pediatrics, Fujita Health University School of Medicine, Toyoake, Japan

**Keywords:** Thyroid diseases, Gene expression, Paediatrics

## Abstract

SLC26A4 is a known iodide transporter, and is localized at the apical membrane of thyrocytes. Previously, we reported that SLC26A7 is also involved in iodide transport and that *Slc26a7* is a novel causative gene for congenital hypothyroidism. However, its detailed role in vivo remains to be elucidated. We generated mice that were deficient in *Slc26a7* and *Slc26a4* to delineate differences and associations in their roles in iodide transport. *Slc26a7*^−/−^ mice showed goitrous congenital hypothyroidism and mild growth failure on a normal diet. *Slc26a7*^−/−^ mice with a low iodine environment showed marked growth failure. In contrast, *Slc26a4*^−/−^ mice showed no growth failure and hypothyroidism in the same low iodine environment. Double-deficient mice showed more severe growth failure than *Slc26a7*^−/−^ mice. RNA-seq analysis revealed that the number of differentially expressed genes (DEGs) in *Slc26a7*^−/−^ mice was significantly higher than that in *Slc26a4*^−/−^ mice. These indicate that SLC26A7 is more strongly involved in iodide transport and the maintenance of thyroid function than SLC26A4.

## Introduction

Congenital hypothyroidism (CH) occurs in 1 in 3000–4000 people^1^, and causes short stature and intellectual disability if left untreated. CH may result from various genetic abnormalities related to thyroid hormone synthesis, thyroid development, and transport of iodide, which is an essential constituent of thyroid hormones^2^.

The transport of iodide into the follicular lumen is essential for thyroid hormone synthesis, but the mechanism of this transport is yet to be fully elucidated. Iodide uptake across the basolateral membrane into the thyroid follicular cells is mediated by the Na^+^/I^−^ symporter (NIS). The iodide from the thyroid follicular cells is transported at the apical membrane into the follicular lumen by SLC26A4 (pendrin)^3^. However, apical iodide efflux at the apical membrane is also possible in the absence of pendrin^4^. This is also supported by the fact that thyroid function is normal in more than half of Pendred syndrome cases caused by SLC26A4 dysfunction, and that *Slc26a4* knockout mice on a low iodine diet show normal thyroid hormone levels^5,6^. This indicates that there are other iodide transporters in the apical membrane of thyroid follicular cells.

In 2019, we reported that SLC26A7, like SLC26A4, was involved in iodide transport at the apical membrane of thyroid follicular cells, and that *Slc26a7* was a novel causative gene for CH^7^. Similarly, Cangul et al. also reported that SLC26A7 dysfunction caused CH, but the function reported for SLC26A7 differed from our findings in some respects^8^. SLC26A7 was first identified as a chloride anion exchanger in the kidneys and stomach^9^. SLC26A7 dysfunction was initially thought to cause renal tubular acidosis and impaired gastric acid secretion in mice^10^. However, it has recently been implicated in CH development as well. The role of SLC26A7 in comparison with SLC26A4 in vivo is still unclear. Therefore, we generated mice genetically modified at the *Slc26a7* and *Slc26a4* loci so as to elucidate the role of these proteins in iodide transport in the thyroid glands.

## Results

### *Slc26a7*^−/−^, but not *Slc26a4*^−/−^ mice, developed goitrous CH

**Figure 1:**
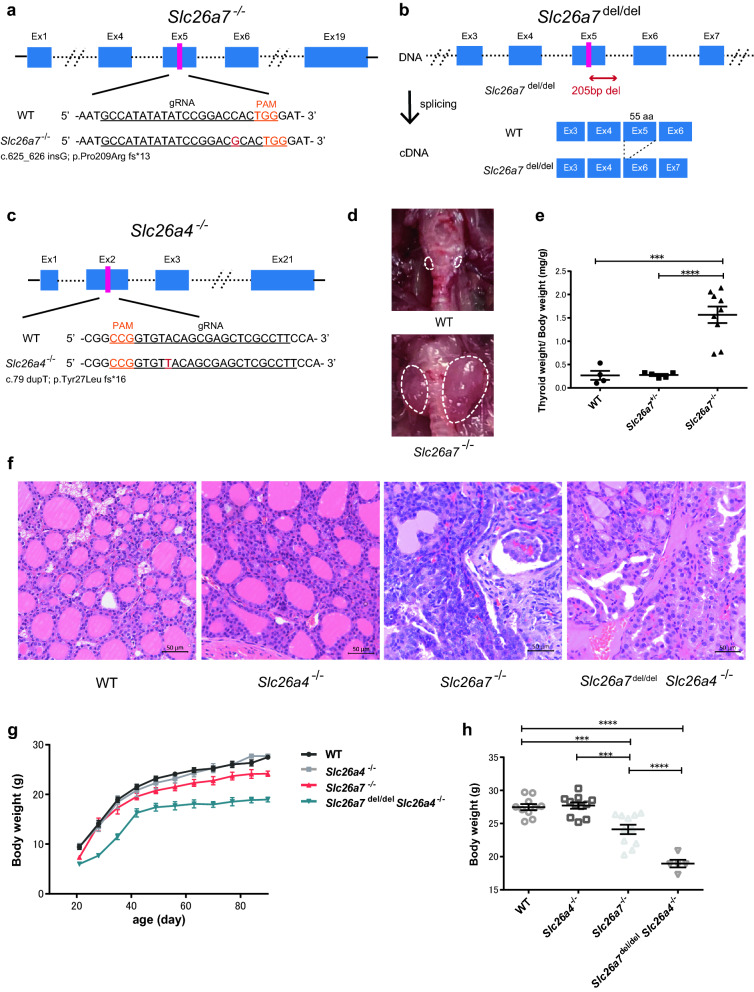
Generation of deficient mice and their auxological and morphological phenotypes. (**a**) Strategy to generate *Slc26a7*^−/−^ mouse. The gDNA target sequence is located in exon 5; black and orange underlining indicates the target sequence and PAM sequence, respectively. In the mutant allele, one base insertion (red letter) is introduced, resulting in a frame shift. (**b**) Strategy to generate *Slc26a7*^del/del^ mouse. The target sequence is the same as in *Slc26a7*^−/−^ mouse, and a 205 bases deletion here causes exon 5 to be skipped when splicing occurs. (**c**) Strategy to generate *Slc26a4*^−/−^ mouse. The target gDNA sequence is located in exon 2. In the mutant allele, one base duplication (red letter) is introduced, resulting in a frame shift. (**d**) Photographic image of the thyroid gland in male wild type (WT) and *Slc26a7*^−/−^ mice at day 90, demonstrating the presence of a goiter in the *Slc26a7*^−/−^ mouse. The area surrounded by dotted lines indicates the thyroid gland. (**e**) Relative weights of the thyroid gland in male WT, *Slc26a7*^+/−^, and *Slc26a7*^−/−^ mice at day 90; n = 4–9 mice of each genotype. One-way analysis of variance (ANOVA) showed significant differences among the three groups (*F* (2, 15) = 24.1, *p* < 0.0001). ***p < 0.001, ****p < 0.0001 determined by Tukey’s test. (**f**) Hematoxylin and eosin-stained thyroid sections from male WT, *Slc26a4*^−/−^, *Slc26a7*^−/−^, and *Slc26a7*^del/del^ *Slc26a4*^−/−^ mice at day 90. (**g**,**h**) Growth curve (**g**) and body weight at day 90 (**h**) of male WT, *Slc26a4*^−/−^, *Slc26a7*^−/−^, and *Slc26a7*^del/del^ *Slc26a4*^−/−^ mice from day 21; n = 5–11 mice of each genotype. One-way ANOVA showed significant differences among the three groups (*F* (3, 33) = 34.3, *p* < 0.0001). ***p < 0.001, ****p < 0.0001 determined by Tukey’s test.

To investigate the function of SLC26A7 and SLC26A4 in the mouse thyroid, we generated mice deficient in these proteins. The targeting constructs for the disruption of *Slc26a7* and *Slc26a4* were designed to create double-strand breaks in exons 5 and 2 of the *Slc26a7* and *Slc26a4* genes, respectively (Fig. 1a–c). We generated heterozygous mutant mice with frameshift variants and crossed them to create deficient mice (*Slc26a7*^−/−^ and *Slc26a4*^−/−^). Genomic DNA and cDNA sequencing confirmed that the frameshift variants were homozygous (Supplementary Fig. S1a,e). Furthermore, qRT-PCR and RNA-seq demonstrated that *Slc26a7*^−/−^ and *Slc26a4*^−/−^ mice showed significantly reduced mRNA expression of the corresponding gene compared to WT mice (Supplementary Fig. S1b,c,f,g). In addition to *Slc26a7*^−/−^ mice, we generated another strain of *Slc26a7*-modified mice, which we designated as *Slc26a7*^del/del^ mice. This strain was found to harbor a 205-base pair deletion that contained exon 5 and the flanking intron in the gene, which resulted in the deletion of exon 5 during splicing (Fig. 1b and Supplementary Fig. S1d). Exon 5 encodes transmembrane segment (TMS) 5, which is crucial for SLC26A7 function^7^. To generate double-deficient mice (*Slc26a7*^del/del^ *Slc26a4*^−/−^), *Slc26a7* ^+/del^ *Slc26a4*^+/−^ female mice were crossed with *Slc26a7*^+/del^ *Slc26a4*^+/−^ male mice.

**Figure 2:**
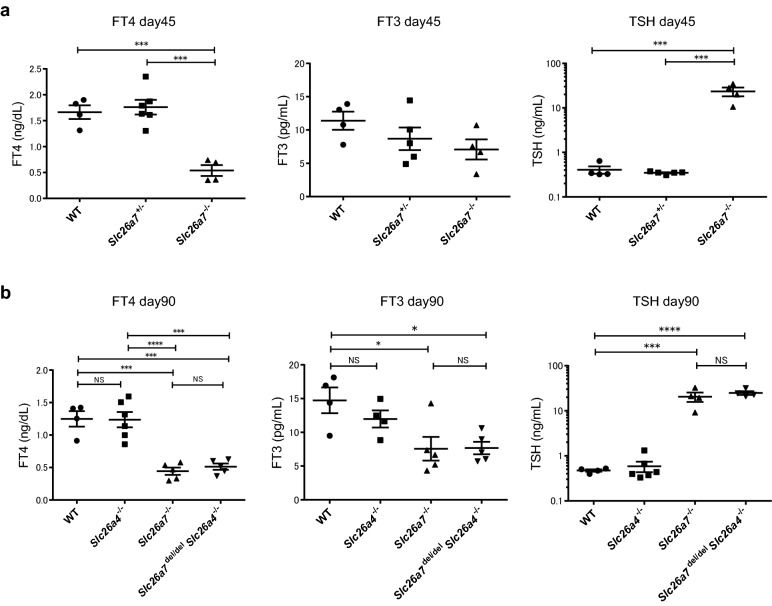
Serological phenotypes of the deficient mice. (**a**) Serum free thyroxine (FT4), free triiodothyronine (FT3), and thyrotropin (TSH) levels in male wild type (WT), *Slc26a7*^+/−^, and *Slc26a7*^−/−^ mice at day 45; n = 4–7 mice of each genotype. One-way analysis of variance (ANOVA) showed significant differences in FT4 and TSH levels (FT4: *F* (2, 11) = 23.4, *p* = 0.0001; TSH: *F* (2, 10) = 22.5, *p* = 0.0002), but not in FT3 levels (*F* (2, 10) = 1.76, *p* = 0.219). ***p < 0.001 determined by Tukey’s test. (**b**) Serum FT4, FT3, and TSH levels in male WT, *Slc26a4*^−/−^, *Slc26a7*^−/−^, and *Slc26a7*^del/del^ *Slc26a4*^−/−^ mice at day 90; n = 4–6 mice of each genotype. One-way ANOVA showed significant differences in FT4, FT3, and TSH levels (FT4: *F* (3, 16) = 22.5, *p* < 0.0001; FT3: *F* (3, 14) = 5.29, *p* = 0.120; TSH: *F* (3, 14) = 30.3, *p* < 0.0001). *p < 0.05, ***p < 0.001, ****p < 0.0001 determined by Tukey’s test. *FT4* free thyroxine, *FT3* free triiodothyronine, *TSH* thyrotropin, *WT* wild type.

Body weights, thyroid sections, and serum FT4, FT3, and TSH levels in WT, *Slc26a7*^−/−^, and *Slc26a4*^−/−^ mice were analyzed by phenotypic analysis. *Slc26a7*^−/−^ mice had evident goiters, and their thyroid weights were significantly higher than those of WT mice (Fig. 1d,e). The characteristic follicular structure was lost in the thyroid glands of *Slc26a7*^−/−^ mice (Fig. 1f). The serum FT4 levels were significantly lower and the serum TSH levels were significantly higher in *Slc26a7*^−/−^ mice than in WT mice (Fig. 2a,b). The serum FT3 levels in *Slc26a7*^−/−^ mice were comparable to those in WT mice at day 45, but were significantly lower at day 90 (Fig. 2a,b). Moreover, *Slc26a7*^−/−^ mice showed mild growth failure compared to WT mice (*Slc26a7*^−/−^: 24.1 ± 0.71 g vs. WT: 27.5 ± 0.46 g; *p* = 0.0008) (Fig. 1g,h). In contrast, *Slc26a4*^−/−^ mice did not show hypothyroidism or growth failure (Figs. 1g, 2b), which is consistent with a previous report^6^. *Slc26a4*^−/−^ mice showed no phenotypic abnormality with reference to the thyroid gland (Figs. 1f, 2b), but showed characteristic degeneration of sensory hair cells in the cochlea (Supplementary Fig. S2a,b)^11^.

There were no differences in thyroid morphology or thyroid hormone levels between *Slc26a7*^−/−^ and *Slc26a7*^del/del^ mice (Supplementary Fig. S3a,b).

### Double-deficient mice showed severe growth failure

We also analyzed double-deficient mice to confirm the phenotype of mice with impairment of both the iodide transporters at the apical membrane of thyroid follicular cells. The size and histology of the thyroid glands of the double-deficient mice were almost identical to those of *Slc26a7*^−/−^ mice (Fig. 1f). Similar to *Slc26a7*^−/−^ mice, the double-deficient mice had significantly lower levels of FT4 and FT3 and significantly higher levels of TSH compared to WT mice (Fig. 2b). When assessed for body weights, the double-deficient mice showed severe growth failure compared to *Slc26a7*^−/−^ mice (double-deficient: 19.0 ± 0.57 g vs. *Slc26a7*^−/−^: 24.1 ± 0.71 g; *p* < 0.0001) (Fig. 1g,h). However, there was no statistically significant difference between *Slc26a7*^−/−^ and double-deficient mice in thyroid function tests. Administering L-T4 to the double-deficient mice tended to improve their growth to some extent (Supplementary Fig. S4a,b).

### Iodine environment greatly influenced the phenotype of *Slc26a7*^−/−^ mice

**Figure 3:**
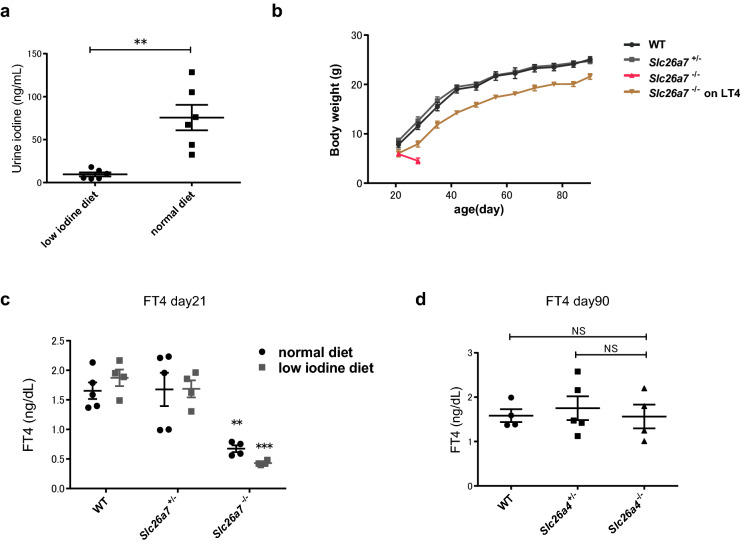
Phenotypes of *Slc26a7*^−/−^ and *Slc26a4*^−/−^ mice on a low iodine diet. (**a**) Urine iodine levels of female *Slc26a7*^+/−^ mice with normal and low iodine diets; n = 6 mice in each group. **p < 0.01 determined by Student’s *t* test. (**b**) Growth curve of male wild type (WT), *Slc26a7*^+/−^, *Slc26a7*^−/−^, and *Slc26a7*^−/−^ mice receiving l-thyroxine (L-T4) replacement from day 21 with a low iodine diet. Note that *Slc26a7*^−/−^ mice without thyroid hormone replacement did not survive. (**c**) Serum free thyroxine (FT4) levels in male WT, *Slc26a7*^+/−^, and *Slc26a7*^−/−^ mice with normal and low iodine diet at day 21; n = 4–5 mice of each genotype. Two-way analysis of variance (ANOVA) showed significant effects of genotype (*F* (2, 20) = 29.92, *p* < 0.0001). **p < 0.01, ***p < 0.001 determined by Tukey’s test compared with WT mice with same diet. (**d**) Serum FT4 levels in male WT, *Slc26a4*^+/−^, and *Slc26a4*^−/−^ mice that were fed a low iodine diet, at day 90; n = 4–5 mice of each genotype. One-way ANOVA showed no significant differences among the three groups (*F* (2, 10) = 0.19, *p* = 0.83). *WT* wild type, *L-T4* l-thyroxine, *FT4* free thyroxine.

To investigate the effect of iodine intake on the phenotypes of *Slc26a7*^−/−^ and *Slc26a4*^−/−^ mice, we fed *Slc26a7*^+/−^ and *Slc26a4*^+/−^ female mice a low iodine diet for 28 days. We found that urine iodine levels were significantly lower in the low iodine diet group (Fig. 3a). Subsequently, these mice were crossed with male mice of the same genotype (*Slc26a7*^+/−^ or *Slc26a4*^+/−^), and their offspring were raised in the same low iodine environment. The genotype ratios of the offspring were consistent with the expected single-locus Mendelian inheritance pattern (*Slc26a7*^+/+^: *Slc26a7*^+/−^: *Slc26a7*^−/−^ = 34: 60: 27; *p* = 0.66, Chi-square test). Unlike WT and *Slc26a7*^+/−^ mice, *Slc26a7*^−/−^ mice showed marked growth failure, and exhibited a 100% mortality rate post-weaning (Fig. 3b). The serum FT4 levels of *Slc26a7*^−/−^ mice at day 21 (at weaning) were significantly lower than those of WT and *Slc26a7*^+/−^ mice (Fig. 3c). Serum FT4 levels in *Slc26a7*^−/−^ mice that were fed a low iodine diet tended to be lower than those in *Slc26a7*^−/−^ mice that were fed a normal diet (Fig. 3c). This suggests that hypothyroidism in *Slc26a7*^−/−^ mice was further aggravated by a low iodine diet. We were able to rescue *Slc26a7*^−/−^ mice that had been raised in a low iodine environment from the fetal stage by administering L-T4 and a high iodine diet after weaning (Fig. 3b). On the other hand, *Slc26a4*^−/−^ mice fed a low iodine diet showed no growth failure or hypothyroidism compared to WT mice (Fig. 3d, Supplementary Fig. S2c).

### *Slc26a7* has a stronger effect on the thyroid phenotype than *Slc26a4*

**Figure 4:**
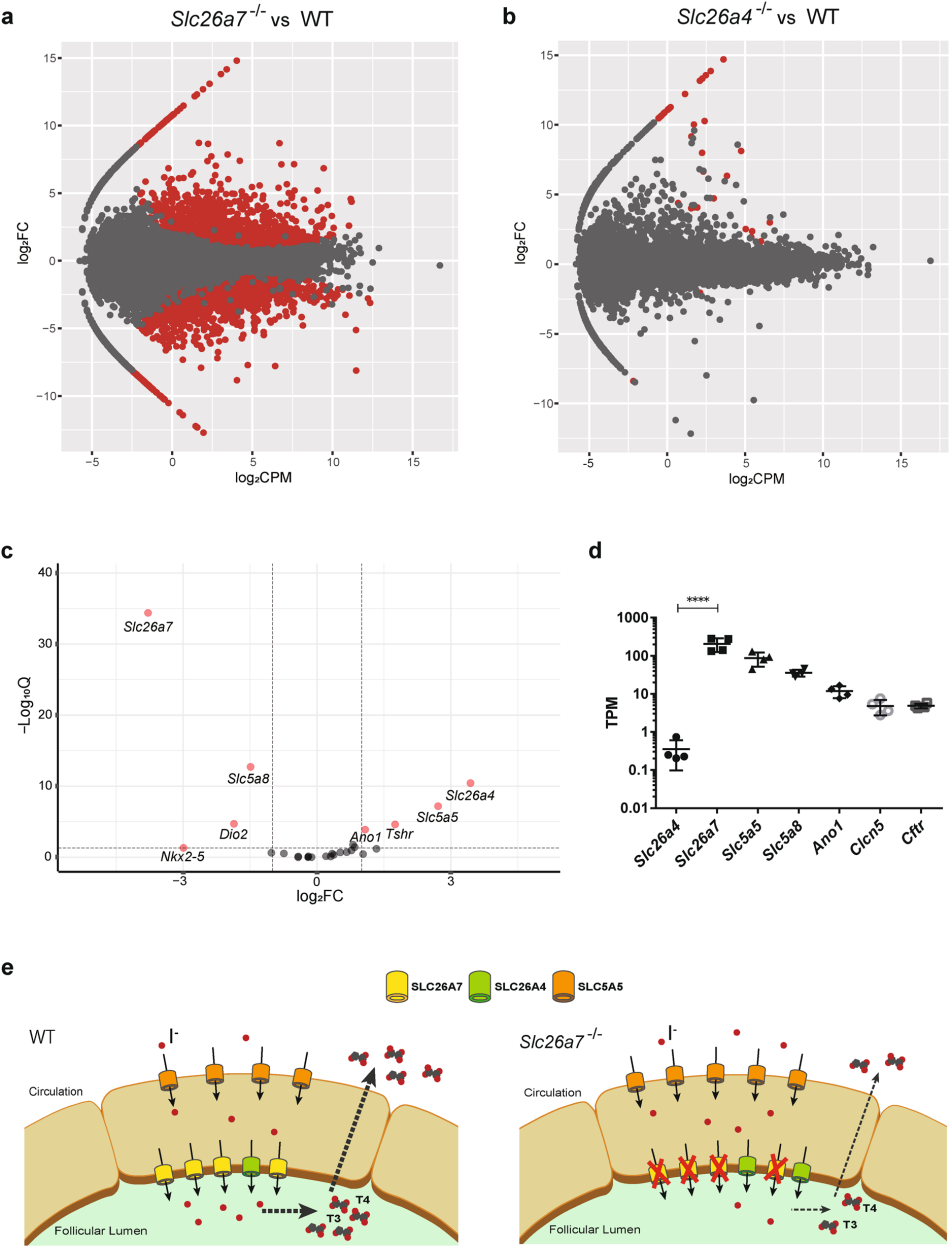
Gene expression profiles were greatly altered in *Slc26a7*^−/−^ mice. (**a**,**b**) Global patterns of gene expression in wild type (WT) (male n = 4), *Slc26a7*^−/−^ (male n = 4), and *Slc26a4*^−/−^ (male n = 3) mice are depicted using a MA plot with counts per million (CPM) and fold change (FC). Genes with a *Q*-value < 0.05 are indicated by red dots. The number of differentially expressed genes (DEGs) between WT and *Slc26a7*^−/−^ was 6371, and that between WT and *Slc26a4*^−/−^ was 37. (**c**) Pattern of expression of genes related to thyroid hormone synthesis is depicted using a volcano plot with FC and *Q*-value. Genes with ≥ 2-fold change and *Q*-value < 0.05 are indicated by pink dots. (**d**) Expression levels of the genes known to encode iodide transporters in male WT mice (n = 4) using the transcripts per million (TPM) value obtained by StringTie. One-way analysis of variance (ANOVA) showed significant differences among the seven groups (*F* (6, 21) = 20.0, *p* < 0.0001). ****p < 0.0001 determined by Tukey’s test. (**e**) Schema of the relationship between *Slc26a7* and *Slc26a4* in the thyroid follicular cell, as deduced from the results of this study. *WT* wild type, *CPM* counts per million, *FC* fold change, *T3* triiodothyronine, *T4* thyroxine, *I*^−^ iodide.

To elucidate the mechanisms of iodide transport in thyroid follicular cells and the cross-talk of the genes related to iodide transport, we compared the gene expression profiles of the thyroid glands of WT, *Slc26a7*^−/−^, and *Slc26a4*^−/−^ mice at day 90 by RNA-seq analysis. First, we analyzed the differentially expressed genes (DEGs) in *Slc26a7*^−/−^ and *Slc26a4*^−/−^ mice in comparison with WT mice. The results showed that *Slc26a7*^−/−^ mice showed far more DEGs compared to *Slc26a4*^−/−^ mice (Fig. 4a,b), suggesting that *Slc26a7* has a stronger effect on the thyroid phenotype than *Slc26a4*. We then analyzed the differential expression of 26 genes involved in thyroid hormone synthesis and release (*Ano1, Cftr, Clcn5, Dio1, Dio2, Dio3, Duoxa1, Duoxa2, Duox1, Duox2, Foxe1, Glis3, Gnas, Iyd, Nkx2-1, Nkx2-5, Pax8, Secisbp2, Slc26a4, Slc26a7, Slc5a5, Slc5a8, Slc16a2,Tg, Tpo, and Tshr*) in *Slc26a7*^−/−^ mice (Supplementary Table 1). The results showed that significant expression changes occurred mainly in the genes involved in iodide transport, with *Slc26a4* being the most upregulated (Fig. 4c). This suggests that SLC26A4 may compensate for the loss of function of SLC26A7. Finally, we quantified the expression levels of the genes encoding iodide transporters (*Slc26a4, Slc26a7, Slc5a5, Slc5a8, Ano1, Cftr, and Clcn5*) using the TPM value obtained by StringTie in WT mice. Results showed that *Slc26a7* expression levels were higher than those of *Slc26a4* (Fig. 4d).

## Discussion

While SLC26A7 deficiency in humans causes goitrous CH, its precise role and the underlying mechanism of CH development are unclear^7,8,12^. In this study, we show that loss of SLC26A7 function in mice leads to goitrous CH (Figs. 1d–f, 2). The serological and morphological findings in *Slc26a7*^−/−^ mice were similar to those in *Slc5a5*-deficient mice^13^. In contrast, *Slc26a4*^−/−^ mice showed characteristic abnormal inner ear findings (Supplementary Fig. S2b), though the thyroid phenotype remained normal. Although *Slc5a5*, *Slc26a4*, and *Slc26a7* have been identified as iodide transporters and genetic abnormalities in these genes are known to cause CH in humans, phenotypic analysis of deficient mice suggests that the effect of *Slc26a4* on the mouse thyroid is less prominent than that of *Slc5a5* and *Slc26a7* under normal conditions.

*Slc26a7*^−/−^ mice fed a low iodine diet showed more severe growth failure than those fed a normal diet (Fig. 3b). This implies that iodine intake greatly influences the phenotype of *Slc26a7*^−/−^ mice. Moreover, all *Slc26a7*^−/−^ mice with a low iodine diet died when therapeutic intervention was not provided; this is presumably due to marked hypothyroidism based on the lower FT4 levels (Fig. 3c), but FT3 levels could not be evaluated at this time.

We have described the differences in the function of SLC26A7 and SLC26A4 in the mouse thyroid based on serological and growth data, and have also demonstrated such differences by RNA-seq analysis. We found that the number of DEGs in *Slc26a7*^−/−^ mice was clearly higher than that in *Slc26a4*^−/−^ mice (Fig. 4a,b). It is well known that the expression of *Slc26a4*, Slc5a5, and *Tshr* is positively regulated by TSH^14–16^, and the expression of these genes is upregulated in *Slc26a7*^−/−^ mice by elevated levels of TSH. Nevertheless, among the genes involved in thyroid hormone synthesis, *Slc26a4* was identified as the most upregulated gene in *Slc26a7*^−/−^ mice (Fig. 4c), suggesting that SLC26A4 might have a compensatory relationship with the function of SLC26A7 at the apical membrane of thyroid follicular cells (Fig. 4e). However, we were unable to validate the RNA-seq results by qRT-PCR analysis. Furthermore, it would be more informative if a similar genetic analysis could be performed on *Slc26a7*^−/−^ mice treated with L-T4 to normalize the TSH levels, as the results would reflect only the direct effects of SLC26A7 deficiency. These issues must be addressed in future studies.

We also generated and analyzed double-deficient mice to verify the relationship and homology between SLC26A7 and SLC26A4. Interestingly, the double-deficient mice showed significant growth failure compared to *Slc26a7*^−/−^ mice (Fig. 1g,h). This finding also implies that despite its low expression level, SLC26A4 may functionally complement SLC26A7, at least partially. The growth failure of the double-deficient mice improved to some extent with the administration of L-T4 (Fig. 1g,h). Therefore, it is possible that hypothyroidism may have contributed to the growth failure. However, contrary to our expectations, there was no difference in the degree of hypothyroidism between *Slc26a7*^−/−^ and double-deficient mice at day 90 (Fig. 2b). Thus, it is possible that SLC26A4 was not able to complement SLC26A7 deficiency with reference to iodide uptake to an extent that could significantly improve thyroid function at day 90. The possible causes of the weight differences between *Slc26a7*^−/−^ and double-deficient mice might include non-thyroidal effects and a complementary role in thyroid function during the pre- and post-weaning periods. We were not able to examine the non-thyroid phenotype of *Slc26a7*^−/−^ and double-deficient mice in this study and cannot rule out the possibility that renal tubular acidosis, which has been reported in the past^10^, may affect growth retardation. Evaluation of thyroid function prior to day 90 and renal tubular function may provide more insight into the impact of SLC26A7 and SLC26A4 on phenotype.

In addition, this study has several limitations. First, although we analyzed *Slc26a7* and *Slc26a4* mRNA, we did not analyze the corresponding proteins due to the lack of specific antibodies. Additionally, the localization of SLC26A7 in the mouse thyroid remains unclear. Second, we could not investigate the pathophysiological mechanism by which functional *Slc26a7* deficiency causes goitrous hypothyroidism. In our previous study, we demonstrated that SLC26A7 is involved in iodide transport in vitro^7^. In vivo data from the present study support the in vitro data, indicating that SLC26A7 is involved in iodide transport, and that there is likely to be cross-talk between SLC26A7 and SLC26A4. However, further mechanistic details could not be obtained. Third, because there is a paucity of clinical information on congenital hypothyroidism caused by loss of function of *SLC26A7*, we do not believe that these results in mice are entirely consistent with the human phenotype.

In conclusion, we revealed that the phenotypes of *Slc26a7*- and *Slc26a4*-deficient mice were distinct. SLC26A7 played a more important role in the mouse thyroid than SLC26A4, while cross-talk between SLC26A7 and SLC26A4 was present.

## Methods

### Animals and treatments

The mice were maintained under specific pathogen-free conditions in a laboratory animal facility at Nagoya City University. All experiments comply with the ARRIVE (Animal Research: Reporting of In Vivo Experiments) guidelines 2.0, were performed in compliance with the relevant Japanese and institutional laws and guidelines, and were approved by the Institutional Animal Care and Use Committee (IACUC) of Nagoya City University School of Medical Sciences (authorization number H30M-42). C57BL/6N mice were purchased from Japan SLC Inc. (Hamamatsu, Japan). *Slc26a7* and *Slc26a4* mutant mice were generated with a C57BL/6N background using the CRISPR/Cas9 system. For Cas9 guide RNA, the target sequences for *Slc26a7* (5′-GCCATATATATCCGGACCAC-3′) and SLC26A4 (5′-GTGTACAGCGAGCTCGCCTT-3′) were selected in exon 5 and exon 2, respectively, and were purchased from Integrated DNA Technology (IDT; Coralville, IA, USA) (Fig. 1a–c, Supplementary Fig. S1). RiboNucleoProtein (RNP) complexes with Alt-R S.p. Cas9 nuclease V3 (IDT) were transferred to C57BL/6N fertilized oocytes as previously described^17^. F0 mice with the desired mutations were crossed with wild-type (WT) mice to obtain heterozygous F1 mice. These heterozygous F1 mice were then crossed with each other to generate single-deficient mice (*Slc26a7*^−/−^, *Slc26a7*^del/del^, and *Slc26a4*^−/−^). To generate *Slc26a7* and *Slc26a4* double-deficient mice (*Slc26a7*^del/del^ *Slc26a4*^−/−^), double heterozygous mice with in-frame deletions in *Slc26a7* (*Slc26a7*^+/del^) and frameshift insertions in *Slc26a4* (*Slc26a4*^+/−^) were created and intercrossed. Genotyping was performed using the Sanger sequencing technique. Genomic DNA was extracted from tail biopsies using the KAPA MG kit (Kapa Biosystems, Woburn, MA), and analyzed using a SeqStudio genetic analyzer (ThermoFisher Scientific, Waltham, MA). The primer sequences used are listed in Supplementary Table 1.

Blood samples were collected from the retroorbital plexus of mice anesthetized with isoflurane; thyroid samples were collected after the mice had died from prolonged anesthesia with isoflurane. Mice were routinely fed a standard chow containing approximately 1.7 mg Iodine (I)/kg (CE-2; CLEA Japan, Tokyo, Japan). Female *Slc26a7*^+/−^ and *Slc26a4*^+/−^ mice were fed low iodine chow containing less than 500 µg I/kg (CLEA Japan) for analyzing the phenotypes associated with a low iodine diet. After 28 days, the mice were placed in metabolic cages and urine iodine was measured in 24-h urine samples. These mice were bred with *Slc26a7*^+/−^ or *Slc26a4*^+/−^ male mice. The same iodine environment was maintained during pregnancy and postpartum. Thyroid hormone replacement with albuminized drinking water containing 0.39 mg/L l-thyroxine (L-T4) (L-T4 sodium salt pentahydrate; Med Chem Express, NJ) was started after weaning on day 21. In the case of *Slc26a7*^−/−^ mice with a low iodine diet, in addition to providing water containing L-T4, subcutaneous injections of 40 ng/g body weight L-T4 were administered on day 21, 24, and 28; these mice were also fed a high iodine chow containing about 11.9 mg I/kg (CLEA Japan).

### Measurement of serum hormones

Total blood samples were centrifuged (3000*g*) to separate the serum. Serum free thyroxine (FT4), free triiodothyronine (FT3), and thyrotropin (TSH) concentrations were determined by enzyme-linked immunosorbent assay according to the manufacturer’s instructions (FT4: Mouse Free Thyroxine ELISA Kit CSB-E05080m, CUSABIO, Houston, TX, USA; FT3: Mouse free tri-iodothyronine ELISA kit CSB-E05077m, CUSABIO; TSH: rodent TSH ELISA test kit ERKR7015, Endocrine Technologies, Newark, CA, USA). The lowest detectable limits were 0.30 ng/dL, 1.30 pg/mL, 0.20 ng/mL for FT4, FT3 and TSH, respectively. Additionally, the intra-assay variations for each assay were < 15%, < 15%, < 6% for FT4, FT3 and TSH, respectively.

### Determination of urine iodine

Urine iodine concentrations in mice were determined by inductively coupled plasma mass spectrometry (ICP-MS) (ELAN DRC II, Perkin Elmer, Waltham, MA, USA). Sample and standard solutions were prepared by alkaline digestion with 25% tetramethyl ammonium hydroxide (TMAH). Iodine was detected at mass ^127^I, with ^130^Te as the internal standard.

### Thyroid histology

For histological analysis, thyroid glands were fixed overnight in 10% neutralized buffered formalin (NBF), washed with 1 × PBS, and embedded in paraffin. The sections were then stained with hematoxylin and eosin using standard procedures.

### Fluorescence confocal microscopy of cochlea

The cochlear structures were dissected, fixed in 4% paraformaldehyde, and permeabilized in Triton X-100 for 15 min. The tissues were stained with fluorescein-conjugated phalloidin (Thermo Fisher Scientific) in blocking solution (1% bovine serum albumin in PBS) for 60 min. After several washes in PBS, the samples were mounted and examined using a laser scanning confocal microscope (A1RS+, Nikon, Tokyo, Japan).

### RNA-Seq analysis

Thyroid glands were collected from male WT (n = 4), *Slc26a7*^−/−^ (n = 4), and *Slc26a4*^−/−^ (n = 3) mice on day 90. RNA was extracted using the RNeasy Plus Mini Kit (QIAGEN, Hilden, Germany). Libraries were prepared using the TruSeq Stranded mRNA Library Prep Kit (Illumina, San Diego, CA, USA), and sequenced with 151-nt paired-end reads on the NovaSeq 6000 system (Illumina).

Adapter sequences and low-quality (quality-value < 20) bases were trimmed from the 3′-ends of the RNA-seq reads using Trim Galore v0.6.4 (https://www.bioinformatics.babraham.ac.uk/projects/trim_galore/). The processed reads were mapped onto the mouse genome version mm10 using HISAT2 v2.1.0 software^18^. From the mapping data, the expression of individual genes was quantified with StringTie v2.1.1^19^, using the mm10 gene annotation obtained from the University of California at Santa Cruz (UCSC) Genome Browser^20^. The analyses of differential gene expressions of the *Slc26a7*^−/−^ and *Slc26a4*^−/−^ groups with respect to WT were performed using edgeR v3.32.1 (https://www.ncbi.nlm.nih.gov/pmc/articles/PMC2796818/) implemented in R v.4.0.3 (https://www.R-project.org/); qvalue package (https://rss.onlinelibrary.wiley.com/doi/abs/10.1111/1467-9868.00346) was used to correct the *p*-values for multiple comparisons. Counts Per Million (CPM) and Transcripts Per Million (TPM) values were computed using StringTie.

### qRT-PCR analysis

RNA was extracted from the thyroid tissue using the method described earlier, and 500 ng of total RNA was used for cDNA synthesis using SuperScript IV Reverse Transcriptase (Invitrogen, Carlsbad, CA). The cDNA was amplified using the FastStart Essential DNA Green Master Mix (Roche, Mannheim, Germany). All procedures were as per the manufacturer’s protocols. A Light Cycler 96 system (Roche) was used for amplification and a large intron was selected. Primers were designed to be located in the exons before and after that intron (see Supplementary Table 2 for primer sequences). Each sample was analyzed in triplicate under the same conditions. The standard curve was prepared by making a stepwise dilution of the cDNA of the sample to be measured. The relative quantification was performed by taking into account the PCR efficiency obtained from the standard curve, and the data were normalized to the GAPDH internal control.

### Statistical analysis

Statistical comparisons were performed using the Student’s *t* test, Chi-square test, and one-way analysis of variance (ANOVA), followed by Tukey’s test for post hoc analysis (GraphPad Prism 6.0, GraphPad Software, San Diego, CA). A *p*-value < 0.05 was considered significant. Data are shown as mean ± SEM.

## Supplementary Information

Supplementary Information.

## Data Availability

The authors confirm that the data supporting the findings of this study are available within the article. In addition, the raw RNA-sequencing data were deposited in the DNA Data Bank of Japan Sequence Read Archive (https://www.ddbj.nig.ac.jp/dra/index-e.html; accession numbers: DRR377288–DRR377298).
