# Supplementary material for: The iodide transporter Slc26a7 impacts thyroid function more strongly than Slc26a4 in mice
Source: Sci Rep. 2022 Jul 4;12:11259. doi: 10.1038/s41598-022-15151-4 (PMC9253019; doi:10.1038/s41598-022-15151-4)
Supplement: Supplementary file 1 — Supplementary Information. [file 41598_2022_15151_MOESM1_ESM.docx]

**The iodide transporter *Slc26a7* impacts thyroid function more strongly than *Slc26a4* in mice**

Naoya Yamaguchi, Atsushi Suzuki, Aya Yoshida, Tatsushi Tanaka, Kohei Aoyama, Hisashi Oishi, Yuichiro Hara, Tomoo Ogi, Izuki Amano, Satomi Kameo, Noriyuki Koibuchi, Yasuhiro Shibata, Shinya Ugawa, Haruo Mizuno, and Shinji Saitoh

**Supplementary Informatio
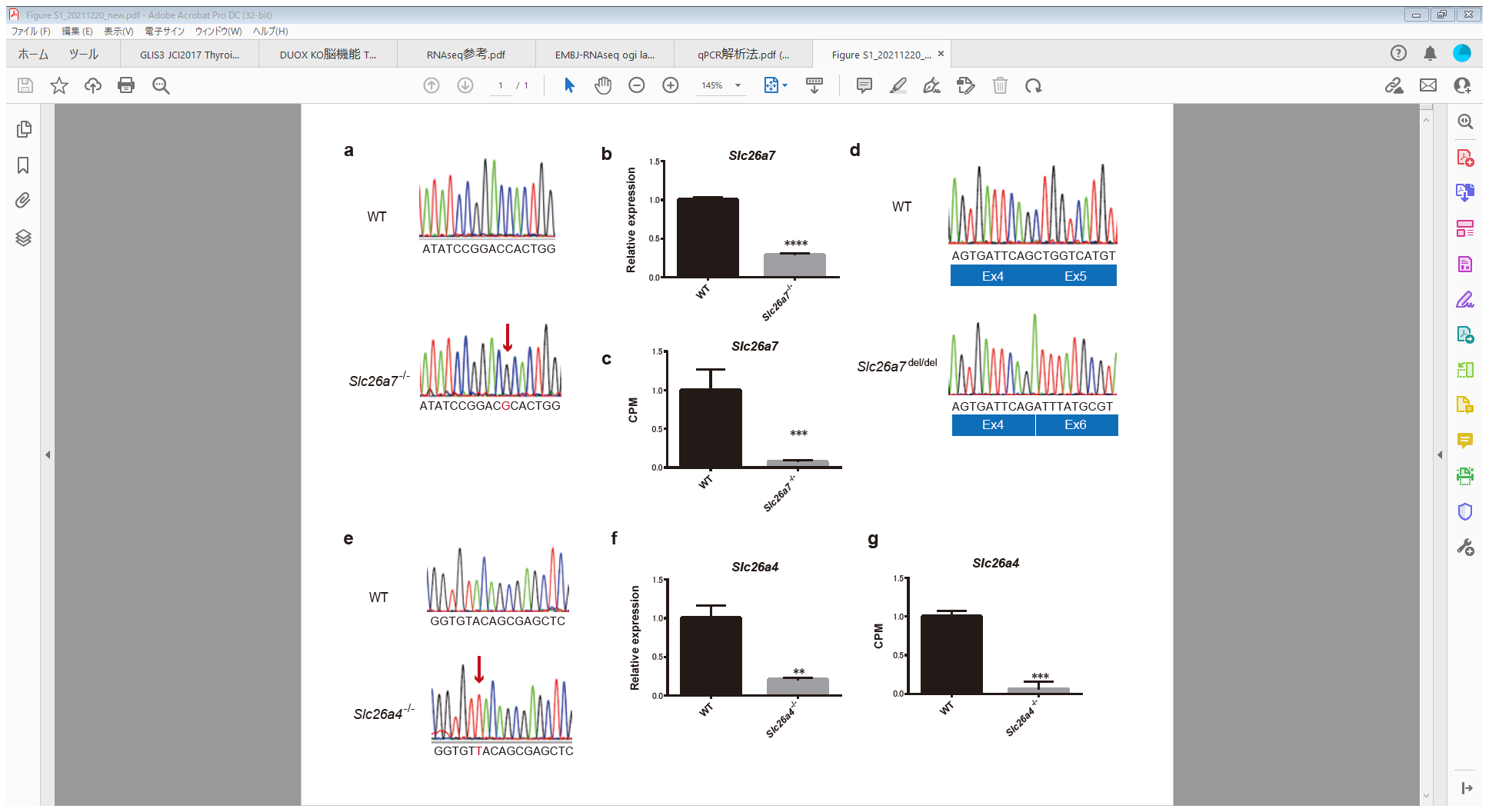
n**

**Supplementary Figure S1. Generation of deficient mice, and their genotypes**

(a) Genomic DNA sequence chromatograms of a *Slc26a7*^-/-^ mouse show a single base insertion (red letter and arrow) that caused a frame shift.

(b) Relative expression of *Slc26a7* in the thyroid glands from wild type (WT) and *Slc26a7*^-/-^ mice was analyzed by qRT-PCR; n = 3 mice of each genotype. *****p* < 0.0001 determined by Student’s t-test.

(c) Expression levels of *Slc26a7* in male WT mice (n = 4) and *Slc26a7*^-/-^ (n = 4) mice using the counts per million (CPM) value obtained using StringTie. ****p* < 0.001 determined by Student’s t-test.

(d) Complementary DNA sequence chromatograms of a *Slc26a7*^del/del^ mouse indicate that the skipping of exon 5 has occurred.

(e) Genomic DNA sequence chromatograms of a *Slc26a4*^-/-^ mouse show a single base duplication (red letter and arrow) that caused a frame shift.

(f) Relative expression of *Slc26a4* in the thyroid glands from wild type (WT) and *Slc26a4*^-/-^ mice was analyzed by qRT-PCR; n = 3 mice of each genotype. ***p* < 0.01 determined by Student’s t-test.

(g) Expression levels of *Slc26a4* in male WT mice (n = 3) and *Slc26a4*^-/-^ (n = 3) mice using the counts per million (CPM) value obtained using StringTie. ****p* < 0.001 determined by Student’s t-test.

WT, wild type

**Supplementary Figure S2. Phenotype of *Slc26a4*^-/-^ mice**

(a, b) Inner ear findings in *Slc26a4*^+/-^ and *Slc26a4*^-/-^ mice. In *Slc26a4*^-/-^ mice, the degeneration of sensory hair cells in the cochlea was observed.

(c) Body weights of wild type (WT), *Slc26a4*^+/-^, and *Slc26a4*^-/-^ mice fed a low iodine diet at day 90; n = 5-6 mice of each genotype. One-way analysis of variance (ANOVA) showed no significant difference among the three groups (*F* (2, 13) = 0.023, *p* = 0.97). WT, wild type

**Supplementary Figure S3. Phenotypes of *Slc26a7* ^del/del^ mice**

(a) Hematoxylin and eosin-stained thyroid sections from male *Slc26a7*^del/del^ mice at day 90.

(b) Serum free thyroxine (FT4) and thyrotropin (TSH) levels in male wild type (WT), *Slc26a7*^-/-^, and *Slc26a7*^del/del^ mice at day 90; n = 4-5 mice of each genotype. One-way analysis of variance (ANOVA) showed significant differences in FT4 and TSH (FT4: *F* (2, 10) = 35.0, *p* < 0.0001; TSH: *F* (2, 9) = 12.15, *p* = 0.0028). **p* < 0.05, ***p* < 0.01, ****p* < 0.001, *****p* < 0.0001 determined by Tukey’s test.

WT, wild type; FT4, free thyroxine; NS, not significant


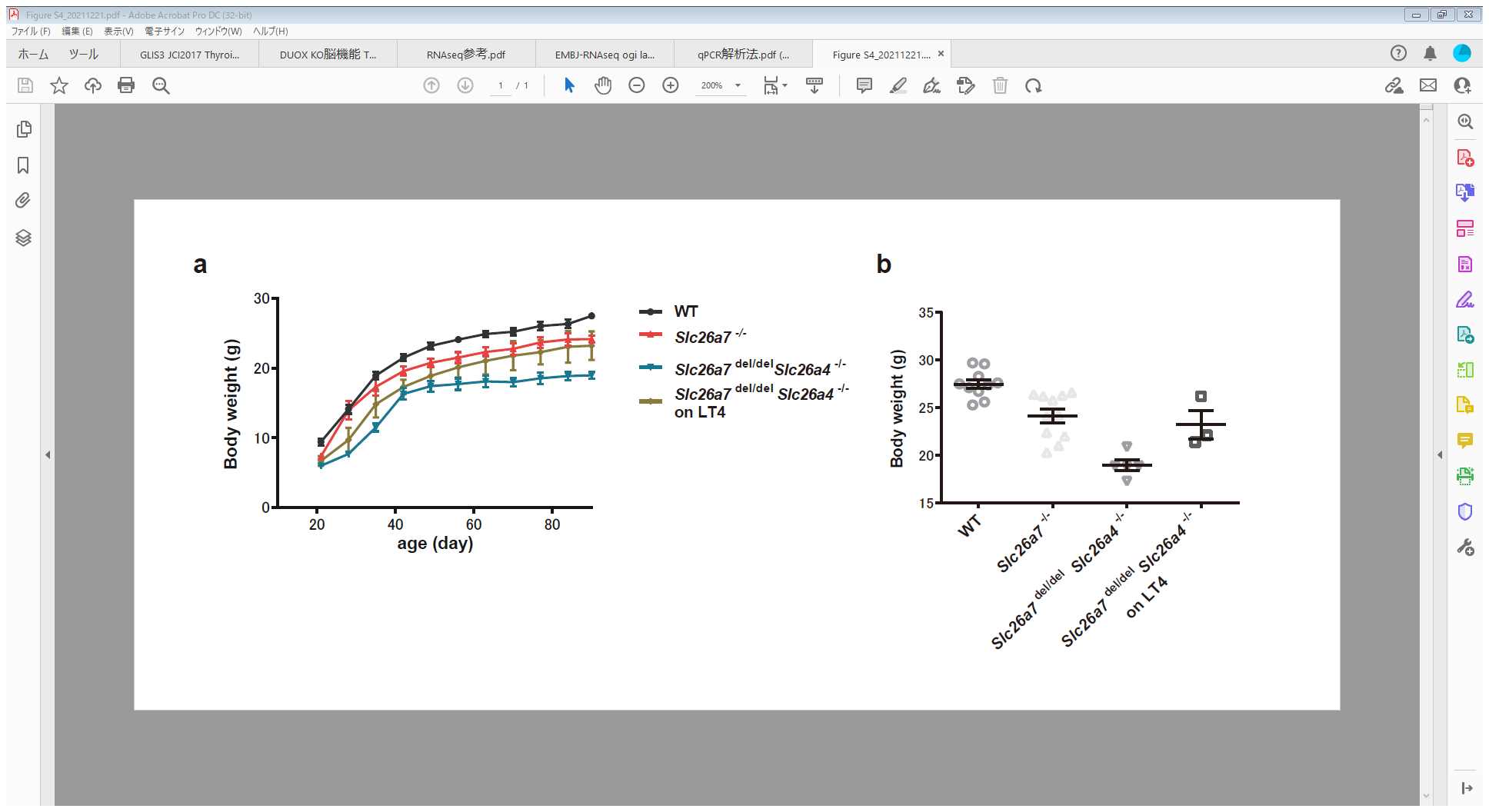


**Supplementary Figure S4. Body weights of *Slc26a7*^del/del^ *Slc26a4*^-/-^ mice on L-thyroxine (L-T4)**

(a, b) Growth curves from day21 (a) and body weights at day 90 (b) of male WT, *Slc26a7*^-/-^, *Slc26a7*^del/del^ *Slc26a4*^-/-^ mice and *Slc26a7*^del/del^ *Slc26a4*^-/-^ mice receiving L-T4 ; n = 3-11 mice of each genotype.

L-T4, L-thyroxine

**Supplementary Table 1.** Results of the differential expression of 26 genes involved in thyroid hormone synthesis and release

| gene | P-value | Q-value | log FC | log CPM | CPM  WT 1 | CPM  WT 2 | CPM  WT 3 | CPM  WT 4 | CPM  *Slc26a7*^-/-^ 1 | CPM  *Slc26a7*^-/-^ 2 | CPM  *Slc26a7*^-/-^ 3 | CPM  *Slc26a7*^-/-^ 4 |
| --- | --- | --- | --- | --- | --- | --- | --- | --- | --- | --- | --- | --- |
| ***Slc26a7*** | 7.09E-38 | 4.13E-35 | -3.785525 | 7.2034691 | 216.95570 | 324.14041 | 206.88519 | 351.27967 | 15.6887961 | 16.050713 | 19.430319 | 28.531074 |
| ***Slc5a8*** | 3.61E-15 | 1.93E-13 | -1.489044 | 6.2201275 | 98.052822 | 121.51386 | 122.45377 | 97.625771 | 35.6692369 | 41.114241 | 36.468567 | 43.360652 |
| ***Slc26a4*** | 1.09E-12 | 3.71E-11 | 3.4392452 | 1.8832033 | 0.4161158 | 0.3879133 | 0.4590223 | 1.2083245 | 9.78778438 | 5.9875980 | 6.345279 | 4.7786807 |
| ***Slc5a5*** | 3.53E-09 | 6.62E-08 | 2.7131207 | 9.1536337 | 245.55259 | 135.09083 | 154.22194 | 68.000394 | 1093.85322 | 1220.2401 | 699.39075 | 939.76282 |
| ***Dio2*** | 1.84E-06 | 2.01E-05 | -1.859651 | 4.2995385 | 16.095713 | 16.757857 | 25.992139 | 64.546814 | 6.56905073 | 10.880013 | 7.3188932 | 9.2283375 |
| ***Tshr*** | 2.29E-06 | 2.44E-05 | 1.7501197 | 7.7075352 | 96.963838 | 81.296944 | 38.022350 | 166.86876 | 360.953649 | 384.07703 | 250.36993 | 293.50500 |
| ***Ano1*** | 1.46E-05 | 0.0001294 | 1.0779044 | 5.6119670 | 28.499506 | 38.442214 | 40.298335 | 18.484795 | 64.7694232 | 66.420146 | 54.438465 | 79.756965 |
| ***Nkx2-1*** | 0.0036854 | 0.0170457 | 0.8063386 | 8.1100645 | 190.22690 | 259.2619 | 171.51178 | 183.07403 | 352.532308 | 348.52847 | 336.84534 | 368.23260 |
| ***Glis3*** | 0.0094129 | 0.0380552 | 0.8437894 | 5.8222453 | 23.798283 | 43.368714 | 29.559125 | 65.180970 | 86.1871603 | 85.531984 | 34.647237 | 84.230123 |
| ***Nkx2-5*** | 0.0123446 | 0.0476545 | -2.990941 | -2.510945 | 0.5312116 | 0.0775826 | 0.4590223 | 0.0599877 | 0 | 0.0807921 | 0.0251796 | 0.0313356 |
| ***Tpo*** | 0.0178183 | 0.0650825 | 1.3333574 | 11.264793 | 1547.0831 | 1349.8803 | 1057.4535 | 1637.9911 | 4807.67466 | 3390.6322 | 2591.9121 | 3302.0135 |
| ***Pax8*** | 0.0338048 | 0.1102053 | 0.7719422 | 8.9250217 | 362.82643 | 407.68725 | 214.90852 | 450.70507 | 649.536400 | 563.29203 | 549.29468 | 690.16684 |
| ***Dio1*** | 0.0670496 | 0.1927510 | 0.6658245 | 8.9860822 | 507.80295 | 428.28545 | 303.146 | 329.12705 | 709.659915 | 733.77250 | 505.76741 | 538.97251 |
| ***Clcn5*** | 0.0741281 | 0.2089914 | 0.5211217 | 3.7811942 | 7.8265187 | 15.545628 | 9.9454839 | 11.791876 | 19.2111838 | 16.086620 | 10.676183 | 18.762197 |
| ***Duox1*** | 0.0873421 | 0.2376332 | -1.022266 | 0.7368922 | 1.1686656 | 0.8243159 | 1.8456523 | 4.9961222 | 1.33607811 | 0.4039608 | 1.2925568 | 1.3160956 |
| ***Gnas*** | 0.1150199 | 0.2946945 | -0.742455 | 10.493202 | 1803.0918 | 2113.6041 | 1715.0222 | 1585.2104 | 1131.14195 | 1112.1222 | 1066.4601 | 1003.9851 |
| ***Duoxa2*** | 0.1201987 | 0.3046890 | 0.3396951 | 5.7860437 | 64.312027 | 48.062466 | 37.706772 | 44.699440 | 73.8385595 | 66.572753 | 54.942058 | 51.155385 |
| ***Dio3*** | 0.1504458 | 0.3630831 | 1.0339041 | -1.233571 | 0.4515299 | 0.0678848 | 0.3920815 | 0.1542542 | 0.51621199 | 0.5027068 | 0.3609087 | 0.806892 |
| ***Foxe1*** | 0.2138301 | 0.4732596 | 0.3737243 | 6.5300052 | 114.38758 | 97.977219 | 45.777915 | 63.826961 | 149.337094 | 121.37678 | 65.878432 | 80.595192 |
| ***Cftr*** | 0.3963081 | 0.7319801 | 0.2010399 | 4.2763062 | 23.948793 | 15.274088 | 18.781663 | 14.045702 | 20.4966529 | 20.036459 | 23.584965 | 18.70736 |
| ***Secisbp2*** | 0.4120503 | 0.7412656 | -0.191921 | 3.9586926 | 16.387880 | 17.950691 | 17.079456 | 14.834112 | 13.0874924 | 12.998563 | 15.603007 | 16.302351 |
| ***Duoxa1*** | 0.4428687 | 0.7786391 | -0.419150 | 0.7105104 | 1.1598121 | 0.8825029 | 1.1953706 | 4.1648634 | 1.22473827 | 0.9066677 | 1.5947129 | 1.8096315 |
| ***Iyd*** | 0.4649350 | 0.802926 | 0.3068667 | 9.6381660 | 919.89041 | 795.61031 | 510.49977 | 623.62403 | 989.720108 | 938.88588 | 863.96513 | 732.46991 |
| ***Slc16a2*** | 0.6279400 | 0.9661620 | -0.170904 | 8.8493271 | 437.16950 | 575.13005 | 378.38741 | 563.28493 | 503.094141 | 483.36164 | 310.43186 | 438.80040 |
| ***Duox2*** | 0.6402448 | 0.9778858 | -0.120821 | 4.5368853 | 25.383064 | 27.706712 | 18.466086 | 25.109156 | 22.9360076 | 23.654154 | 13.127005 | 29.181288 |
| ***Tg*** | 0.68390357 | 1 | -0.421541 | 16.681137 | 95268.467 | 148404.34 | 79606.866 | 158016.729 | 108860.699 | 81834.655 | 58187.324 | 110465.849 |

WT, wild type; FC, fold change; CPM, counts per million

**Supplementary Table 2.** List of primers used in this study

| Primers used for sequencing of genomic DNA from the deficient mice | | |
| --- | --- | --- |
| *Slc26a7* | Forward | 5’-TGATGCCCAACTCTCCTC-3’ |
| *Slc26a7* | Reverse | 5’-TCTCACCAAACACCCAAC-3’ |
| *Slc26a4* | Forward | 5’-TTTCCGAACGTCATCCCT-3’ |
| *Slc26a4* | Reverse | 5’-ACTCTCACTACTCCATCAA-3’ |
|  |  |  |
| Primers used for sequencing of complementary DNA from the deficient mice | | |
| *Slc26a7* (for *Slc26a7*^-/-^ mouse) | Forward | 5’-GCTGTTTCTTTCTTGGGT-3’ |
| *Slc26a7* (for *Slc26a7*^-/-^ mouse) | Reverse | 5’-AGGCGGCAATGATCAAAA-3’ |
| *Slc26a7* (for *Slc26a7*^del/del^ mouse) | Forward | 5’-CTTTCATCTGTACACCCA-3’ |
| *Slc26a7* (for *Slc26a7*^del/del^ mouse) | Reverse | 5’-CCCAACTACTTCTAATCC-3’ |
| *Slc26a4* | Forward | 5’-TTTCCGAACGTCATCCCT-3’ |
| *Slc26a4* | Reverse | 5’-CCGAACACAAAATACGTCA-3’ |
|  |  |  |
| Primers used for qRT-PCR | | |
| *Slc26a7* | Forward | 5’-AAAGGAAAGTGACAGCAAC-3’ |
| *Slc26a7* | Reverse | 5’-GACAGGGAACTGAGCAAG-3’ |
| *Slc26a4* | Forward | 5’-AGAACCAGGTCAAATCCA-3’ |
| *Slc26a4* | Reverse | 5’-TTCATCTCTGCCTCCATC-3’ |
